# Night Temperature Determines the Interannual Yield Variation in Hybrid and Inbred Rice Widely Used in Central China Through Different Effects on Reproductive Growth

**DOI:** 10.3389/fpls.2021.646168

**Published:** 2021-06-04

**Authors:** Liying Huang, Fei Wang, Yi Liu, Yunbo Zhang

**Affiliations:** ^1^Engineering Research Center of Ecology and Agricultural Use of Wetland, Ministry of Education, Yangtze University, Jingzhou, China; ^2^College of Agriculture, Yangtze University, Jingzhou, China; ^3^Minister of Agriculture Key Laboratory of Crop Ecophysiology and Farming System in the Middle Reaches of the Yangtze River, College of Plant Science and Technology, Huazhong Agricultural University, Wuhan, China; ^4^Hubei Collaborative Innovation Center for Grain Industry, Yangtze University, Jingzhou, China

**Keywords:** rice, grain yield, climate variability, night temperature, grain filling percentage

## Abstract

Interannual variation in grain yield of rice has been observed at both farm and regional scales, which is related to the climate variability. Previous studies focus on predicting the trend of climate change in the future and its potential effects on rice production using climate models; however, field studies are lacking to examine the climatic causes underlying the interannual yield variability for different rice cultivars. Here a 6-year field experiment from 2012 to 2017 was conducted using one hybrid (Yangliangyou6, YLY6) cultivar and one inbred (Huanghuazhan, HHZ) cultivar to determine the climate factors responsible for the interannual yield variation. A significant variation in grain yield was observed for both the inbred and hybrid cultivars across six planting years, and the coefficient of variation for grain yield was 7.3–10.5%. The night temperature (average daily minimum temperature, T_min_) contributed to the yield variability in both cultivars. However, the two cultivars showed different responses to the change in T_min_. The yield variation in HHZ was mainly explained by the effects of T_min_ on grain filling percentage and grain weight, while the change in spikelets m^−2^ in response to T_min_ accounted for the yield variability in YLY6. Further analysis found that spikelets m^−2^ of YLY6 significantly and negatively correlated with T_min_ from transplanting to heading. For HHZ, the grain filling percentage and grain weight were significantly affected by T_min_ of the week prior to heading and from heading to maturity, respectively. Overall, there were differences in the response mechanism between hybrid and inbred cultivars to high night temperature. These will facilitate the development of climate-resilient cultivars and appropriate management practices to achieve a stable grain yield.

## Highlights

- Night temperature determines the inter-annual yield variation of hybrid and inbred cultivars.
- Hybrid and inbred cultivars showed different responses to the change in T_min_.
- T_min_ affected spikelets m^−2^ of hybrid rice, and grain filling and grain weight of inbred rice.

## Introduction

Rice is the staple food for more than half of the population in the world (Tao et al., 2014). China is the largest rice producer and consumer, producing 28.1% of the world rice output using 18.8% of the global rice-growing area (FAO, 2016). In the next decade, rice production must increase by 20% to meet the growing demand for food in China (Huang and Zou, 2018). As the arable land area decreases year by year due to the rapid development of urbanization, increasing rice yield per unit area has become an effective way to increase rice production (An et al., 2018). Yield potential is defined as the yield of a cultivar when grown in environments to which it is adapted, with nutrients and water nonlimiting and with pests, diseases, weeds, lodging, and other stresses effectively controlled (Evans, 1993). The maximum yield potential of irrigated rice reaches as high as 15.0 t ha^−1^ in China because of the development of super hybrid cultivars through the combination of interspecific heterosis and ideotype, and adopting the optimized management practices (Cheng et al., 2007; Peng et al., 2008; Sun et al., 2013). Nevertheless, yield gap analyses find that the current average Chinese rice yield is approximately 82% of the national yield potential, indicating that Chinese rice yields have been approaching their biophysical potential ceiling (Van Wart et al., 2013). The threshold of 80% is often used as an indicator for yield stagnation (Cassman et al., 2003), because there exists a fundamental constraint for further increasing yield, which appears to be the uncertainty in growing season weather (Lobell et al., 2009). Therefore, identifying the causal environmental factors contributing to the yield variation across planting years would provide the breeding target in the future to further reduce the yield gap in rice.

Variation in grain yield for the same cultivar has been widely observed across locations and planting years (Gravois and Helms, 1998; Wang et al., 2004; Gong et al., 2008; Blanche and Linscombe, 2009; Ma et al., 2011; Jaruchai et al., 2018). The fluctuation in the average yield at the regional scale was also observed; for example, the average yield of single-season rice is 8.27 and 7.95 t ha^−1^ in 2015 and 2016, respectively, in Hubei Province, China (National Bureau of Statistics, n.d.). Multiplying the planting area, the reduction in rice production of Hubei Province in 2016 is around 0.42 Mt, which corresponds to 41.2% of total rice production of the year in the United States (FAOSTAT).^1^ Globally, the average rice yield variability (standard deviation) is about 0.5 tons ha^−1^ year^−1^ (or 13% of average rice yields), and year-to-year climate variability explains about 32% of the rice yield variability (Ray et al., 2015). Among the environmental factors, understanding crop responses to temperature and the magnitude of regional temperature changes are two of the most important needs for the adaptation of agriculture to climate variability (Lobell and Burke, 2008). Projections using climate models suggest that both the increase in global mean temperature and the occurrence of extreme seasonal heat will cause significant damage to rice production (Lobell and Burke, 2008; Battisti and Naylor, 2009; Tao and Zhang, 2013; Zhao et al., 2017). In addition, precipitation and radiation also account for the year-to-year variability in rice yields (Peng et al., 2004; Lobell and Burke, 2008; Tao et al., 2013).

Rice grain yield is comprised of the number of spikelets m^−2^ (panicles m^−2^×spikelets panicle^−1^), grain filling percentage, and grain weight (Zhang et al., 2013). Among them, the number of spikelets m^−2^ is regarded as the most important factor affecting rice yield (Kropff, 1994). Genetic improvement in the yield potential of rice is mainly ascribed to the significant increase in the number of spikelets per panicle in China (Zhu et al., 2016). Newly developed hybrid and super hybrid cultivars have larger panicles and fewer tillers (Huang et al., 2011). In the tropics, the yield advantage of hybrid rice over inbred rice is also accounted for by the larger panicles (Bueno et al., 2010). However, the increased panicle size does not substantially improve yield if there is no sufficient substrate supply to fully satisfy the increased sink demand (Ohsumi et al., 2010). Previous studies indicate that anthesis and ripening are the most temperature-sensitive stages in rice, so spikelets per panicle, grain filling percentage, and grain weight are usually affected by the high temperature at these stages (Tashiro and Wardlaw, 1991; Matsui and Omasa, 2002; Peng et al., 2004; Mohammed and Tarpley, 2009; Sanchez et al., 2014). For example, by analyzing the relationship between rice yield and temperature using data from irrigated field experiments conducted at the International Rice Research Institute Farm from 1992 to 2003, Peng et al. (2004) found that grain yield declined by 10% for each 1°C increase in growing-season minimum temperature in the dry season of tropical rice. Therefore, to combat with the challenges of climate variability on rice production, it is necessary to ascertain the regional environmental factor responsible for interannual yield variability and its physiological mechanisms and then develop climate-resilient cultivars (Mittler and Blumwald, 2010).

Extensive studies have been conducted to predict the trend of climate change in the future and its potential effects on rice production using climate models and investigate the effects of environmental factors on rice yield in controlled environments. However, relatively fewer studies attempt to determine the relationship between the yield variation across years and the corresponding environmental conditions for different rice cultivars in field experiments. In the present study, a 6-year experiment from 2012 to 2017 was conducted with the representative inbred and hybrid rice cultivars in Central China. Rice production in this area accounts for 54% of national rice production in 2017 (National Bureau of Statistics, n.d.). The objectives of the study were to (1) determine the degree of interannual variation in grain yield for the inbred and hybrid cultivars, (2) identify the environmental factors contributing to inter-annual variation in rice grain yield in Central China, and (3) examine the agronomic traits that could be used for developing climate-resilient rice.

## Materials and Methods

### Geographic Region Description

Field experiments from 2012 to 2017 were conducted in Wuxue County (29°51′ N 115°33′ E), Hubei Province, China. Wuxue County is located in the basin of the Yangtze River of Central China and represents a typical agricultural region of this region. The soil in the experimental field was a clay loam with pH of 5.2–5.5, organic C of 23.8–26.7 g kg^−1^, total N of 1.80–2.10 g kg^−1^, available P of 21.3–30.1 mg kg^−1^, and available K of 118.6–158.3 mg kg^−1^.

### Experimental Design and Crop Management

In each growing season, Yangliangyou6 (YLY6) and Huanghuazhan (HHZ) were arranged in a randomized complete-block design with four replications. YLY6 is an *indica* hybrid variety developed by Lixiahe Institute of Agricultural Sciences with Guangzhan63-4 s as the female parent and Yangdao6 as the male parent using the two-line method. HHZ is an *indica* inbred variety developed using the pedigree method with Huangxinzhan as the female parent and Fenghuazhan as the male parent by the Guangdong Academy of Agricultural Sciences in 2007. YLY6 and HHZ are the representative and widely planted cultivars of hybrid and inbred cultivars for single-season rice in Central China, respectively. Pre-germinated seeds were sown and raised in wet seedbeds. Seedlings of 26–31 days were transplanted on 12 June 2012, 10 June 2013, 11 June 2014, 10 June 2015, 13 June 2016, and 17 June 2017, respectively. Hill spacing was 13.3 cm × 30.0 cm with two seedlings per hill in 6 experiment years. Plot size was 30 m^2^ (5 m × 6 m). Nitrogen fertilizer of 180 kg N ha^−1^ as urea was applied in each plot at the basal, tillering, and panicle initiation stages at a ratio of 4:3:3. The amount of N applied by local farmers was 180–200 kg N ha^−1^ for single-season rice. P (40 kg P ha^−1^ as monocalcium phosphate), K (50 kg K ha^−1^ as potassium oxide), and Zn (5 kg Zn ha^−1^ as zinc sulfate) were applied and incorporated in all plots 1 day before transplanting. Additional 50 kg K ha^−1^ as topdressing was applied at the panicle initiation stage. Fertilizer management practices remained consistent from 2012 to 2017. The fields were flooded from transplanting until 10 days before maturity except that the water was drained at the maximum tillering stage to reduce unproductive tillers. Insects, weeds, and diseases were intensively controlled by chemicals to avoid biomass and yield loss. Detailed information about crop management is shown in Supplementary Figure S1.

### Measurement of Grain Yield and Yield Attributes

Twelve hill plants from each plot were sampled at heading and mature stages. After recording the number of panicles, the plant samples were separated into leaves, stems (culm plus sheath), and panicles. The dry weights of each organ except for panicles at maturity were determined after oven-drying at 80°C to constant weight. Panicles at maturity were hand-threshed, and then filled spikelets were separated from unfilled spikelets by submerging them in tap water. After that, empty spikelets were separated from half-filled spikelets by winnowing. Three subsamples of 30-g filled spikelets and 2-g empty spikelets, and total half-filled spikelets were taken to quantify the number of spikelets per m^2^. Dry weights of rachis and filled, half-filled, and empty spikelets were determined after oven drying at 80°C to constant weight. Total aboveground biomass was the summation of leaves, stems, rachis, and filled, half-filled, and empty spikelets dry weight. Spikelets per panicle, grain-filling percentage (100 × filled spikelets m^−2^/total spikelets m^−2^), sink size (total spikelets m^−2^ × grain weight), and harvest index (100 × filled spikelet weight/total aboveground biomass) were calculated. Grain yield was determined from a 5-m^2^ sampling area in the center of each plot and adjusted to a standard moisture content of 0.14 g H_2_O g^−1^ fresh weight.

### Measurement of Climate Parameters

Climate parameters including daily minimum/maximum temperature and solar radiation were collected from the weather station located within 2 km of the experimental site. A data logger (CR800, Campbell Scientific Inc., Logan, Utah, United States) was used as the measurement and control module. A silicon pyranometer (LI-200, LI-COR Inc., Lincoln, NE, United States) and temperature/relative humidity probe (HMP45C, Vaisala Inc., Helsinki, Finland) were used to measure solar radiation and temperature, respectively.

### Statistical Analysis

Analysis of variance was performed with Statistix 9.0, and means were compared based on the least significant difference (LSD) test at the 0.05 probability level. All figures were constructed using SigmaPlot 12.5.

## Results

### Weather Conditions

**Figure 1:**
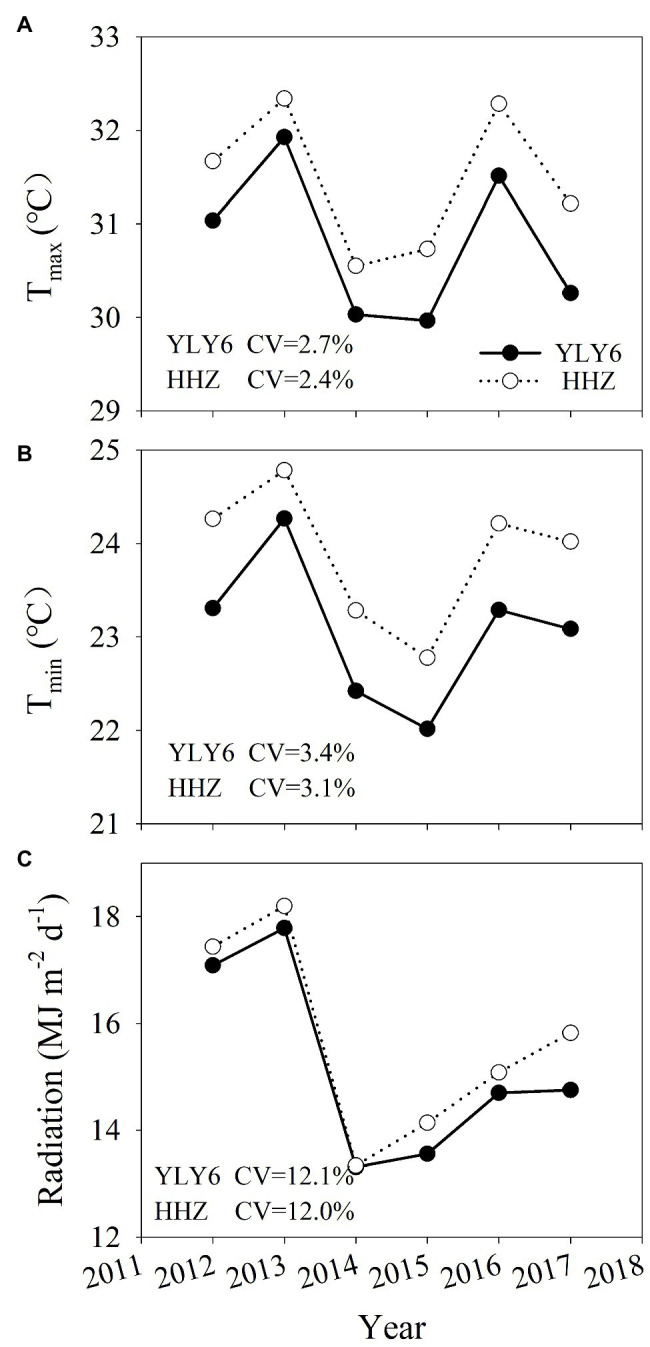
The average daily maximum (T_max_, **A**) and minimum temperature (T_min_, **B**), and solar radiation **(C)** for YLY6 and HHZ during the rice-growing seasons (from transplanting to maturity) in Hubei Province, China, from 2012 to 2017. CV is the coefficient of variation.

The average daily maximum and minimum temperatures were 30.8 and 23.1°C for YLY6 and 31.5 and 23.9°C for HHZ during the rice-growing seasons from 2012 to 2017, respectively (Figure 1). The average annual mean solar radiation was 15.2 and 15.7 MJ m^−2^ d^−1^ for YLY6 and HHZ, respectively. The annual mean daily maximum and minimum temperatures and solar radiation of YLY6 ranged from 30.0 to 31.9°C, 22.0 to 24.3°C and 13.3 to 17.8 MJ m^−2^ d^−1^, and the corresponding ranges of HHZ were 30.6 to 32.3°C, 22.8 to 24.8°C, and 13.3 to 18.2 MJ m^−2^ d^−1^ across years, respectively. The growing-season average temperature and solar radiation were the highest in 2013 and relatively lower in 2014 and 2015. In general, HHZ experienced higher temperature and solar radiation than YLY6 during the rice-growing seasons from transplanting to maturity.

### Growth Duration

**Figure 2:**
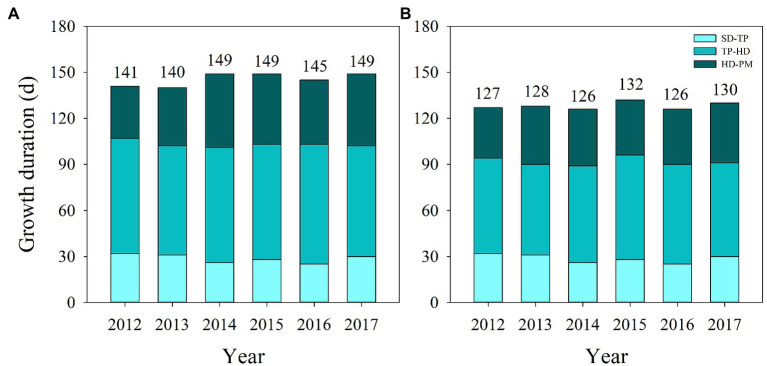
Growth duration of YLY6 **(A)** and HHZ **(B)** from seeding to transplanting, transplanting to heading, and heading to maturity in Hubei Province, China, from 2012 to 2017. The numbers above the columns represent total growth duration from seeding to maturity. SD – seeding; TP – transplanting; HD – heading; PM – maturity.

Significant differences in days to heading and total growth duration were observed in YLY6 and HHZ from 2012 to 2017 (Figure 2). Averaged across 2012–2017, pre-flowering (from transplanting to heading), post-flowering (from heading to maturity), and total growth duration (from seeding to maturity) were longer in YLY6 than those in HHZ by 11, 6, and 18 days, respectively. Pre-flowering, post-flowering, and total growth duration of YLY6 ranged from 71 to 78, 34 to 48, and 140 to 149 days across years, respectively. The difference in total growth duration of YLY6 among experimental years was mainly due to the difference in post-flowering growth duration. The total growth duration of YLY6 was the longest in 2014, 2015, and 2017, but the shortest in 2013. The ranges of pre-flowering, post-flowering, and total growth duration of HHZ were 59–68, 33–39, and 126–130 days in 2012–2017, respectively. The total growth duration of HHZ was the highest in 2015, but the lowest in 2014 and 2016.

### Grain Yield and Yield Components

Experimental year, cultivar, and their interaction significantly affected the grain yield and yield components, except for the effect of cultivar on grain filling percentage and the interactive effect on spikelets per panicle (Table 1). Averaged across all experimental years, the grain yield of YLY6 was significantly higher than that of HHZ by 7.1% (9.99 t ha^−1^ in YLY6 vs. 9.33 t ha^−1^ in HHZ). The yield difference between YLY6 and HHZ was explained by sink size (1181.4 g m^−2^ in YLY6 vs. 1031.0 g m^−2^ in HHZ), which was mainly due to the difference in the grain weight (26.9 mg in YLY6 vs. 18.8 mg in HHZ).

**Table 1:** Grain yield and yield components of YLY6 and HHZ from 2012 to 2017.

Variety	Year	Grain yield (t ha^−1^)	Panicles (m^−2^)	Spikelets (panicle^−1^)	Spikelets (×10^3^ m^−2^)	Grain filling (%)	Grain weight (mg)	Sink size (g m^−2^)
YLY6	2012	9.49	229.5	185.9	42.7	75.8	27.3	1167.2
	2013	9.69	207.3	189.1	39.2	81.1	26.8	1052.0
	2014	10.39	229.3	202.4	46.4	76.5	27.2	1258.8
	2015	11.22	245.2	214.9	52.5	93.2	28.2	1478.6
	2016	9.96	220.8	195.6	43.3	74.9	25.5	1103.2
	2017	9.20	221.9	175.6	38.8	88.4	26.6	1028.9
	**LSD**_**0.05**_	**0.54**	**23.3**	**26.1**	**6.1**	**7.1**	**1.0**	**147.9**
	**Mean**	**9.99 A**	**225.7 B**	**193.9 A**	**43.8 B**	**81.6 A**	**26.9 A**	**1181.4 A**
	**CV (%)**	**7.3**	**5.5**	**7.1**	**11.6**	**9.2**	**3.3**	**14.2**
HHZ	2012	9.51	290.1	167.0	48.4	84.5	19.4	939.6
	2013	7.97	293.0	183.7	53.7	75.3	17.6	943.0
	2014	10.08	355.3	189.1	67.0	84.7	19.8	1328.2
	2015	10.63	305.6	160.5	49.0	91.5	20.3	995.2
	2016	9.28	312.5	174.8	54.6	83.7	18.1	985.7
	2017	8.52	328.7	171.7	56.3	77.6	17.7	994.4
	**LSD**_**0.05**_	**0.66**	**22.3**	**18.9**	**5.2**	**2.5**	**0.3**	**87.1**
	**Mean**	**9.33 B**	**314.2 A**	**174.5 B**	**54.9 A**	**82.9 A**	**18.8 B**	**1031.0 B**
	**CV (%)**	**10.5**	**7.8**	**6.0**	**12.3**	**6.9**	**6.3**	**14.3**
**Analysis of variance**								
Year (Y)		∗∗	∗∗	∗	∗∗	∗∗	∗∗	∗∗
Variety (V)		∗∗	∗∗	∗∗	∗∗	ns	∗∗	∗∗
Y*V		∗∗	∗∗	ns	∗∗	∗∗	∗∗	∗

Within a column, means followed by different letters are significantly different between YLY6 and HHZ at *p* ≤ 0.05 according to LSD. ns, not significant at the 0.05 probability level; * and **, significant at the 0.05 and 0.01 probability levels, respectively. In statistical analysis using the “Least Significant Difference” method, LSD0.05 represents the LSD value of the data at P=0.05 probability level. Mean and CV represent the mean value and coefficient of variation of data from 2012 to 2017 for each parameter, respectively.

**Figure 3:**
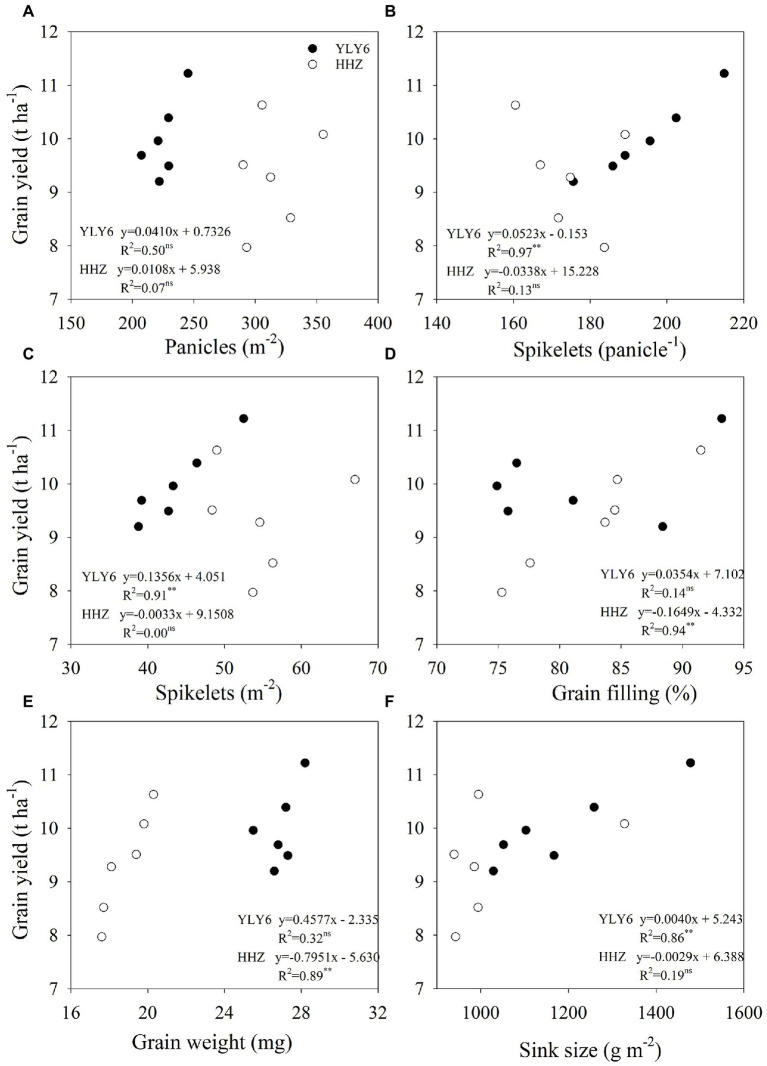
Correlations of yield components (panicles m^−2^, spikelets per panicle, spikelets m^−2^, grain filling percentage, grain weight, and sink size) with grain yield **(A–F)** for YLY6 and HHZ (*n* = 6), respectively. ns and ∗ ∗ are not statistically significant at the *p* ≤ 0.05 and significant at the *p* ≤ 0.01 probability level according to the Student’s *t* test, respectively.

In 6 experiment years, the grain yield of YLY6 ranged from 9.20 to 11.22 t ha^−1^, and the coefficient of variation (CV) was 7.3% (Table 1). The highest and lowest yields of YLY6 were achieved in 2015 and 2017, respectively. All yield components of YLY6 were the highest in 2015 among 6 experiment years, but the minimum value of each yield component was obtained in different years. The CV of yield components for YLY6 varied from 3.3 to 14.2%. Significant positive correlations between grain yield of YLY6 and its spikelets panicle^−1^, spikelets m^−2^, and sink size were observed during the experimental years, and the determinant coefficients (R^2^) were 0.97, 0.91, and 0.86, respectively (Figure 3). The grain yield of HHZ varied from 7.97 to 10.63 t ha^−1^ in 2012–2017, and the CV was 10.5% (Table 1). HHZ produced more than 10 t ha^−1^ of grain yield in 2014 and 2015, but less than 8 t ha^−1^ in 2013. Yield components of HHZ were significantly affected by experiment years, and the CV of yield components ranged from 6.0 to 14.3%. The yield difference of HHZ was mainly explained by the differences in grain filling percentage (*R*^2^ = 0.94) and grain weight (*R*^2^ = 0.89) among experiment years (Figure 3).

### Dry Matter Accumulation and Harvest Index

**Figure 4:**
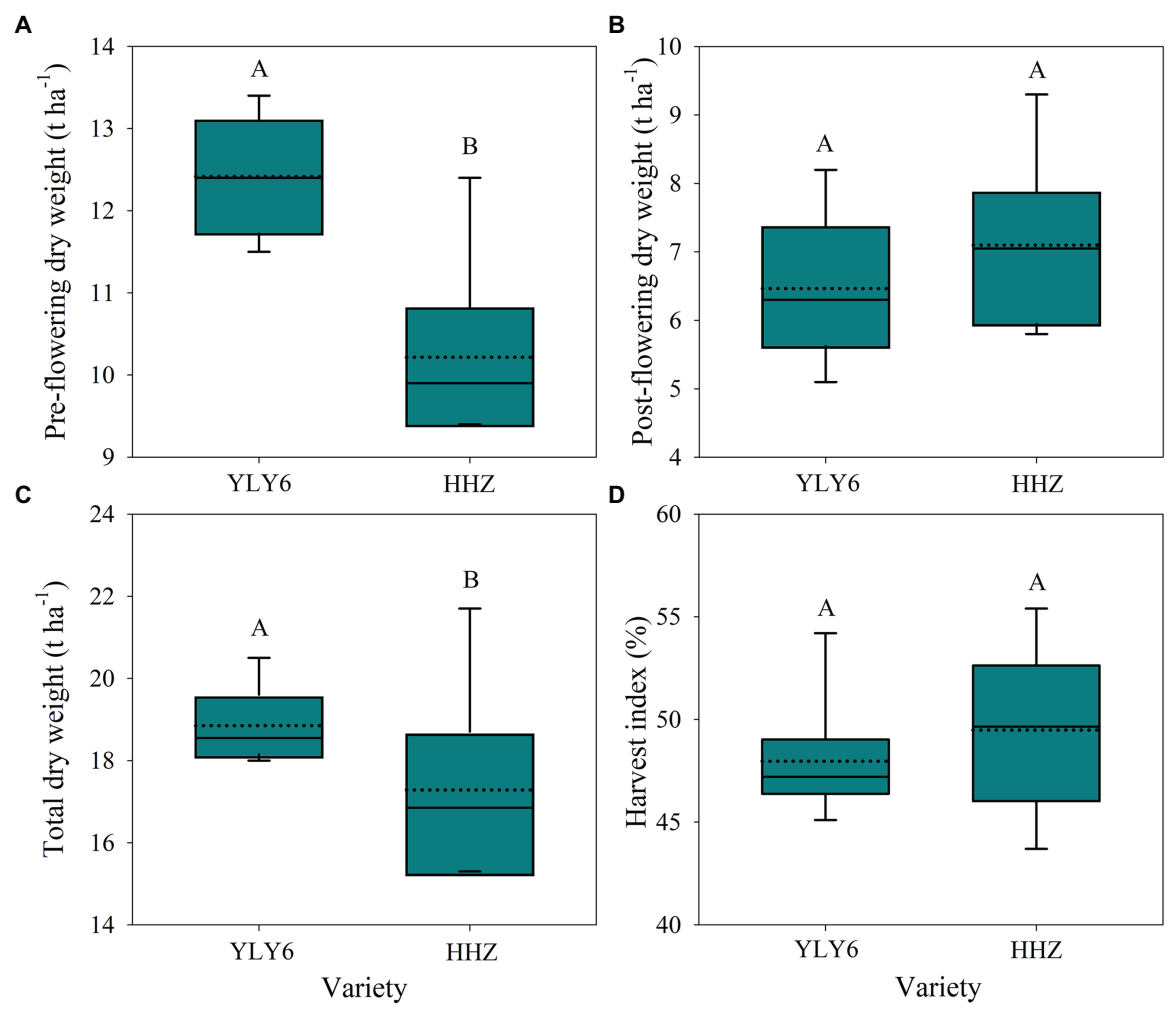
Dry weight before flowering **(A)**, dry weight after flowering **(B)**, total dry weight at maturity **(C)**, and harvest index **(D)** for YLY6 and HHZ in Hubei Province, China, from 2012 to 2017. The box–whisker diagrams show the maximum (top of the vertical line), the 75th percentile (top of the box), the median (horizontal line within the box), the average (dotted lines within the box), the 25th percentile (bottom of the box), and the minimum (bottom of the vertical line) values (*n* = 6). The different letters represent means that are significantly different at the 0.05 probability level according to LSD.

**Figure 5:**
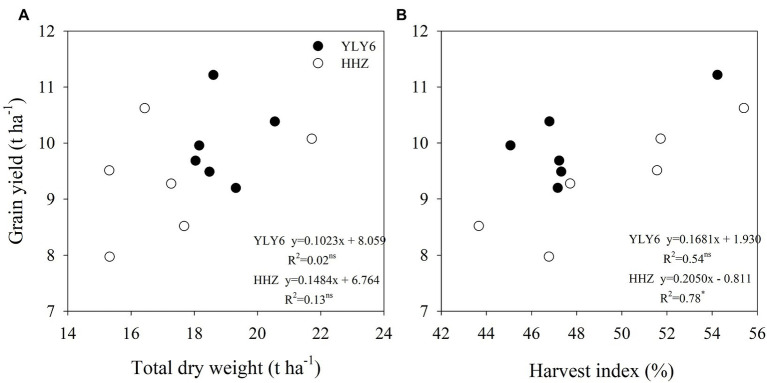
Correlations of total dry weight **(A)** and harvest index **(B)** with grain yield for YLY6 and HHZ (*n* = 6), respectively. ns and ∗ are not statistically significant and significant at the p ≤ 0.05 probability level according to the Student’s t test, respectively.

The total dry weight averaged across 2012–2017 was higher in YLY6 by 9.2% than that in HHZ (Figure 4). A significantly higher total dry weight of YLY6 was mainly attributed to its higher pre-flowering dry weight, since post-flowering dry weight was comparative between the two cultivars. In six experimental years, the total dry weights of YLY6 and HHZ ranged from 18.0 to 20.5 t ha^−1^ and from 15.3 to 21.7 t ha^−1^, and the CV was 5.0 and 13.8%, respectively. The harvest indexes (HI) of YLY6 and HHZ were 48.0 and 49.5% averaged across 2012–2017, respectively, and the difference in the average HI between YLY6 and HHZ was not significant. HI of YLY6 and HHZ varied from 45.1 to 54.2% and from 43.7 to 55.4%, and the CV was 6.6 and 8.5%, respectively. In terms of total dry weight and harvest index, HI rather than total dry weight explained the interannual variation in yield for both YLY6 and HHZ (Figure 5).

### Correlations Between Yield Attributes and Climate Parameters

**Figure 6:**
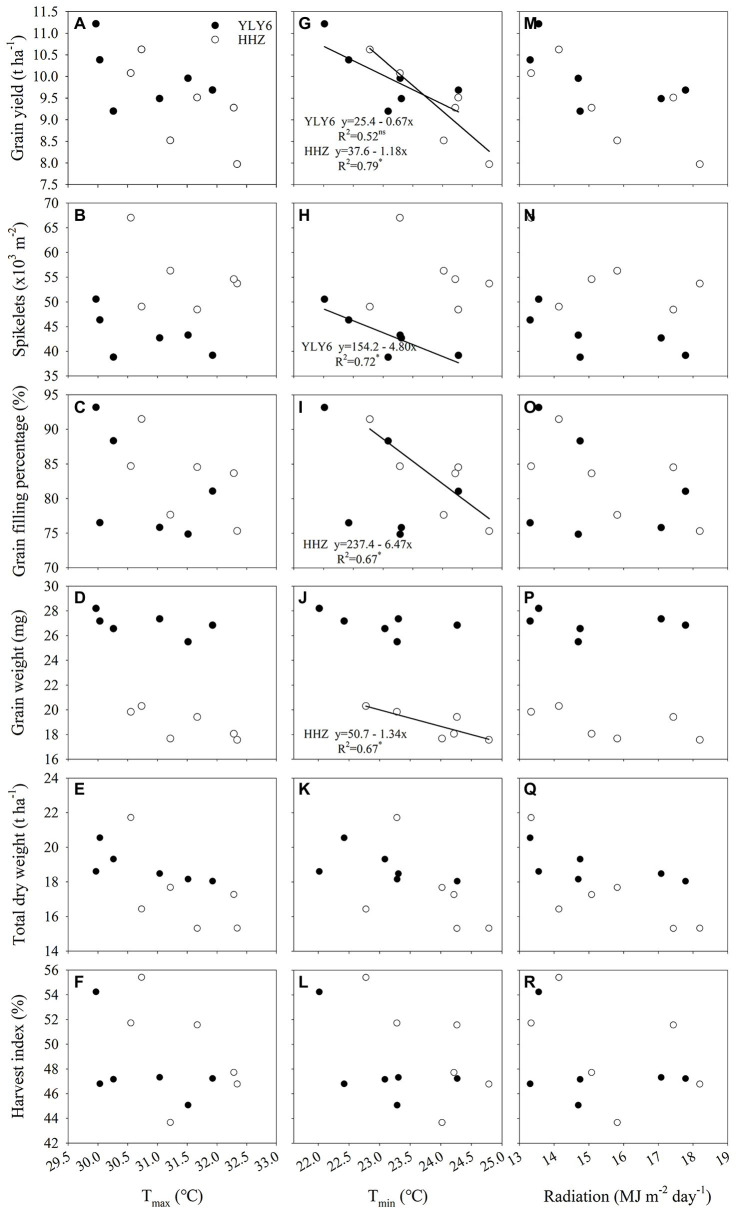
The relationship between rice-yield attributes (grain yield, spikelets m^−2^, grain filling percentage, grain weight, total dry weight, and harvest) and growing-season mean maximum temperature **(A–F)**, minimum temperature **(G–L)**, and radiation **(M–R)** for YLY6 and HHZ. Growing-season mean maximum and minimum temperature and radiation were calculated from daily values for the entire season from transplanting to harvest. ns and ∗ are not statistically significant and significant at the p ≤ 0.05 probability level according to the Student’s t test, respectively. The regression equations are not shown in the graphs when the correlations are not significant. T_max_ – the growing-season mean maximum temperature; T_min_ – the growing-season mean minimum temperature.

Grain yield and yield attributes were not related to the average daily maximum temperature (T_max_) and solar radiation in YLY6 and HHZ (*p* > 0.05; Figure 6). Negative relationships between grain yield and the average daily minimum temperature (T_min_) of the whole growing season were observed in both YLY6 and HHZ, but the correlation was statistically significant only in HHZ (*p* < 0.05; Figure 6G). Around 79% of the interannual yield difference in HHZ was explained by growing-season T_min_. The grain yield of HHZ decreased by 1.18 t ha^−1^ (≈12.6%) for each 1°C increase in the growing-season T_min_. The partial-correlation coefficient between grain yield and minimum temperature with solar radiation held constant was −0.48 and −0.71 for YLY6 and HHZ, respectively. The partial-correlation coefficient between grain yield and solar radiation with minimum temperature held constant was 0.07 and 0.01 for YLY6 and HHZ, respectively (data not shown). The partial-correlation analysis shows that the increases in growing-season T_min_, even small in range, had a negative effect on the grain yield of YLY6 and HHZ, especially on HHZ, and that the effect was independent of solar radiation.

**Figure 7:**
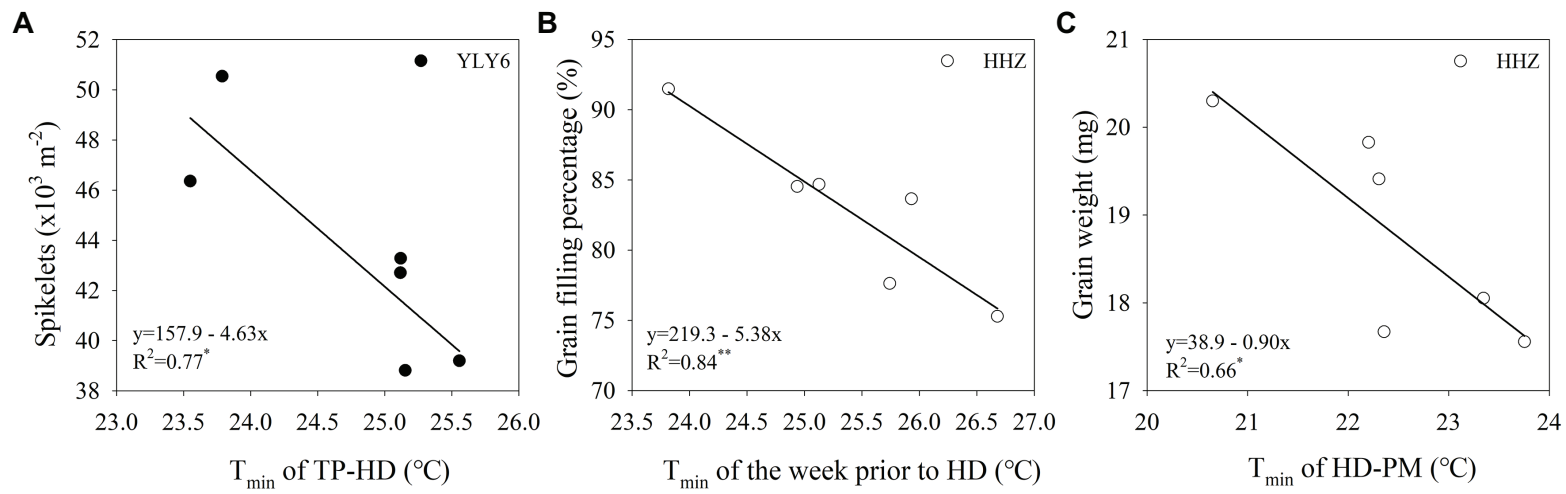
The relationship between spikelets m^−2^ and T_min_ of transplanting (TP) to heading (HD) for YLY6 **(A)**, between grain filling percentage and T_min_ of the week prior to HD for HHZ **(B)**, and between grain weight and T_min_ of HD to maturity (PM) for HHZ (C). * and ** are statistically significant at the *p* ≤ 0.05 and p ≤ 0.01 probability level according to the Student’s t test, respectively. The regression equations are not shown in the graphs when the correlations are not significant. T_min_ – the growing-season mean minimum temperature.

With the increase of growing-season T_min_, spikelets m^−2^ declined linearly in YLY6 (*p* < 0.05; Figure 6H). Spikelets m^−2^ reduced by 13.7 × 10^3^ (≈26.1%) from a 2.3°C increase in minimum temperature for YLY6. There were significant and negative relationships between grain filling percentage, grain weight, and the growing-season T_min_ in HHZ (*p* < 0.05; Figures 6I,J). The growing-season T_min_ explained 67% of the interannual variation in grain filling percentage and grain weight. Panicles m^−2^ were significantly and negatively related to the growing-season T_min_ only in YLY6 (*p* < 0.05), and spikelets panicle^−1^ were not related to the growing-season T_min_ for both YLY6 and HHZ (*p* > 0.05; data not shown). Total dry weight and HI decreased with the increase of the growing-season T_min_, but the correlation was not significant (*p*< 0.05; Figures 6K,L). The average T_min_ from transplanting to maturity was lower in YLY6 than in HHZ, which was mainly due to lower T_min_ from heading to maturity in YLY6 (Supplementary Table S1). Besides, the T_min_ of the week prior to heading and the week post heading were 1.3 and 1.7 lower in YLY6 than those in HHZ averaged across 6 years, respective (Supplementary Table S1). Further analysis found that spikelets m^−2^ of YLY6 significantly and negatively correlated with T_min_ from transplanting to heading. For HHZ, grain filling percentage and grain weight were significantly affected by T_min_ of the week prior to heading and from heading to maturity, respectively (Figure 7).

## Discussion

Rapid increases in minimum night temperature compared to maximum day temperature have been reported and are predicted to continue, posing a big challenge to rice production (Peng et al., 2004). Efforts are required to improve our understanding of the genotypic variation in response to night temperature, which will be conducive to achieving both high and stable yield through genetic improvement. In the present study, significant variation in grain yield was observed for both the inbred and hybrid cultivars across 6 planting years, and the CV for grain yield was 7.3–10.5% (Table 1). The hybrid cultivar YLY6 had a significantly higher and more stable grain yield than the inbred cultivar HHZ. Further analysis illustrated that variability in night temperature contributed to the yield variability in both cultivars despite the larger year-to-year variability in radiation (Figure 6). This is consistent with Wang et al. (2016), who found that the lower rice yield and radiation use efficiency in the tropical environment were mainly due to the higher temperature (instead of radiation) compared with the subtropical environment. By analyzing the historical yields and daily weather data in 1981–2010 on a global scale, Iizumi and Ramankutty (2016) found that over 21% of the yield variability change could be explained by the change in climate variability, especially the variability of temperatures exceeding the optimal range for yield formation. Ray et al. (2015) found that climate variation explained a third of global yield variability, and there were numerous regions where climate variability explained more than 60% of the yield variability in maize, rice, wheat, and soybean. Here we found that variability in night temperature explained around 80% of the interannual yield variability in rice (Figure 6).

Although it is well known that high night temperatures lead to yield loss, few studies focus on the genotypic variation of rice in response to high night temperature, especially in farmers’ field conditions. In the present study, YLY6 (one hybrid cultivar) and HHZ (one inbred cultivar) are both the most widely planted cultivars in the last decades in Central China, which showed different responses to the change in T_min_ (Figures 6, 7). A significant negative correlation between grain yield and T_min_ was observed in HHZ, but not in YLY6 (Figure 6). Yang et al. (2017) reported that YLY6 performed better than HHZ in grain yield under daytime, nighttime, and all-day elevated-temperature treatments. Rehmani et al. (2014) found that Shanyou-63 was more susceptible to daytime warming, while Teyou-559 was affected more by nighttime warming. These studies suggested that significant genotypic variation exists among the elite hybrid and inbred rice cultivars in response to night temperature.

The yield variation in YLY6 was mainly explained by the effect of T_min_ on spikelets m^−2^, but not on grain filling percentage and grain weight (Figure 6). For example, T_min_ was the highest in 2013 during the 6 years, and grain yield of YLY6 was higher than that of HHZ by 1.72 t ha^−1^ (21.6%) in this year (Table 1; Figure 1). Reduction in spikelets m^−2^ of YLY6 was compensated by a high grain filling percentage, which resulted in the high grain yield in 2013 (Table 1). T_min_ had a significant effect on the spikelets m^−2^ of YLY6, but not in HHZ, possibly because of the difference in their spikelets per panicle. YLY6, as a hybrid variety, had more spikelets per panicle than HHZ (Table 1), which might result in more spikelet abortion in YLY6 when exposed to heat stress. The spikelet fertility of rice was extremely sensitive to high temperature, and the adverse effect of high temperature on spikelet fertility was even higher at nighttime than at daytime (Fahad et al., 2018). Mohammed and Tarpley (2009) found that spikelet fertility was significantly reduced due to the reduction in pollen germination when night temperature during the reproductive growth was increased from 27 to 32°C in the greenhouse. The decrease of spikelet fertility in rice caused by high temperature mainly occurred at panicle initiation and flowering stages, which might be related to the development of reproductive organs, the process of anther dehiscence, powder dispersal, and pollen tube elongation (Cheng et al., 2009; Fahad et al., 2018; Hu et al., 2020). In the study, we also found that with the increase of T_min_ from transplanting to heading, the spikelets m^−2^ decreased significantly in YLY6, but not in HHZ (Figure 7). Peng et al. (2004) found that spikelets m^−2^ were significant reduced along with the increase in night temperature. The physiological mechanism of the different responses of spikelets m^−2^ to T_min_ between YLY6 and HHZ requires further investigation.

The change in grain filling percentage and grain weight in response to T_min_ also accounted for the yield variability in HHZ across 6 planting years (Figure 6). In field conditions, a near 2°C increase in night temperature during the reproductive growth resulted in a change in grain filling percentage by −12.2 to +2.7% (Shah et al., 2014). High-temperature exposure during grain filling resulted in a decrease in grain weight due to the limited supply of assimilates and shortened grain-filling duration (Kobata and Uemuki, 2004; Shi et al., 2013). Morita et al. (2005) found that the adverse effect of high temperature on grain weight was greater at nighttime than at daytime. HHZ, as an inbred variety, had shorter post-flowering growth duration and total dry weight than YLY6, which might cause lower grain weight. Besides, high night temperature during the grain filling stage led to increased whole plant senescence and restricting translocation of dry matter from source to sink (Impa et al., 2021), which might have further reduced the grain filling percentage and grain weight in HHZ. Wu et al. (2016) also reported that yield reduction in HHZ was partly attributed to reduction in grain weight. Further analysis illustrated that grain filling percentage and grain weight for HHZ were significantly affected by T_min_ of the week prior to the heading and grain filling stages, respectively (Figure 7). The reproductive growth stage including panicle initiation, flowering, and grain filling is the most temperature-sensitive stage in rice. As a result, T_min_ affected the interannual yield variation in YLY6 and HHZ through different effects on reproductive growth. Despite the significant effect of high night temperature on yield variability through its effects on the development of spikelets and grain filling, the underlying physiological mechanisms are still obscure and need more attention.

Developing climate-resilient cultivars has been proposed and implemented by worldwide scientists to overcome the challenges of climate change (Mittler and Blumwald, 2010; Kole et al., 2015; Harfouche et al., 2019). Here we demonstrate the advantage of hybrid rice over inbred rice in both yield potential and yield stability (Table 1). It has been widely reported that the grain yield of hybrid rice is higher than that of inbred rice (Yuan et al., 1994; Peng et al., 1999, 2008). Given the different responses of grain yield and yield components of hybrid and inbred varieties to T_min_, more attention should be paid to improving spikelet fertility of hybrid rice, and increasing grain filling and grain weight of inbred rice. Therefore, climate-resilient varieties and reasonable cultivation practices could be used to reduce yield loss caused by global warming, especially the increase of night temperature.

## Conclusion

The present study found significant interannual variability in grain yield of hybrid and inbred cultivars in Central China, which was related to the variability in night temperature. The yield variation in HHZ was mainly explained by the effects of T_min_ on grain filling percentage and grain weight, while the change in spikelets m^−2^ in response to T_min_ accounted for the yield variability in YLY6. The genotypic difference was observed in the response of yield and yield components to the change in night temperature. There is a potential for genetic improvement in the yield potential and yield stability simultaneously in response to the increasing climate variability.

## Data Availability Statement

The original contributions presented in the study are included in the article/Supplementary Material, further inquiries can be directed to the corresponding author.

## Author Contributions

YZ conceived and designed the research. LH and YL conducted the experiments and collected the data. LH and FW analyzed the data and wrote the paper. YZ and FW commented and revised the paper. All authors contributed to the article and approved the submitted version.

### Conflict of Interest

The authors declare that the research was conducted in the absence of any commercial or financial relationships that could be construed as a potential conflict of interest.

The reviewer DX declared a shared affiliation, with no collaboration, with one of the authors FW to the handling editor at the time of the review.

## Footnotes

^1^http://www.fao.org/faostat/en/#home
