# Supplementary material for: Night Temperature Determines the Interannual Yield Variation in Hybrid and Inbred Rice Widely Used in Central China Through Different Effects on Reproductive Growth
Source: Front Plant Sci. 2021 Jun 4;12:646168. doi: 10.3389/fpls.2021.646168 (PMC8212977; doi:10.3389/fpls.2021.646168)
Supplement: Supplementary file 1 [file Data_Sheet_1.pdf]

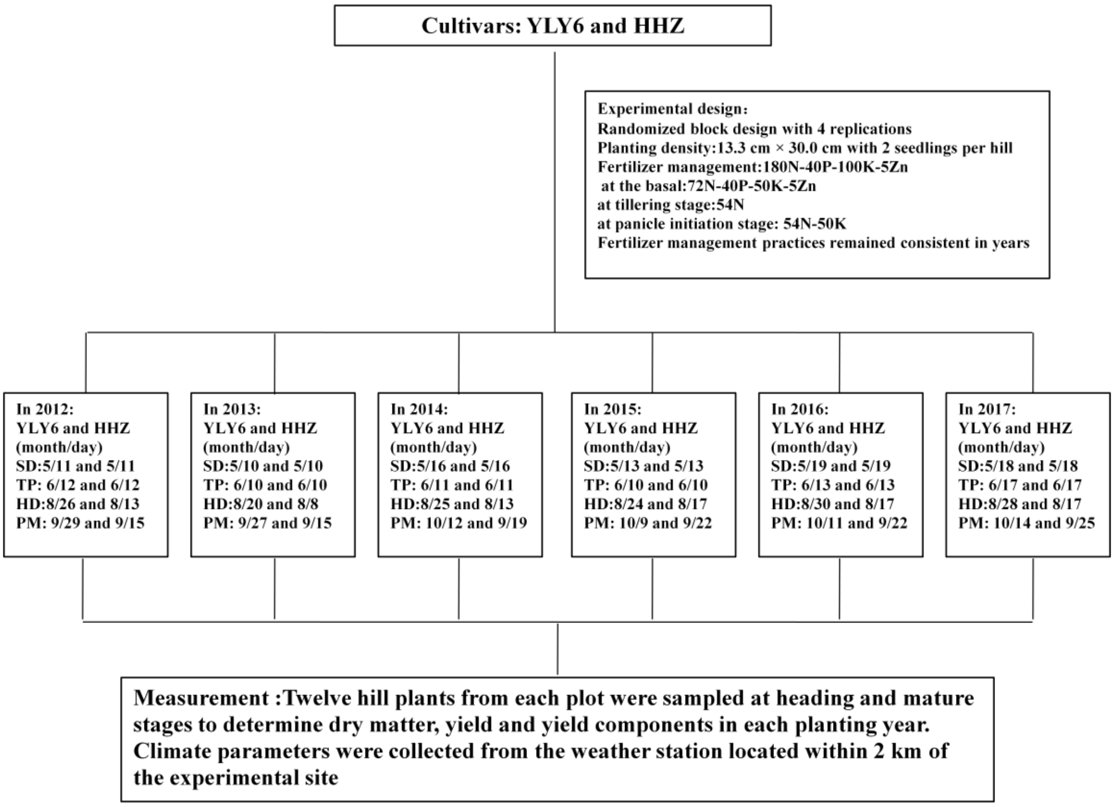

Figure S1 The schematic diagram of experimental densign and crop management for 2012-2017. YLY6, Yangliangyou6; HHZ, Huanghuazhan; SD, sowing date; TP, transplanting; HD, heading, PM, physiological maturity.

Table S1 The daily minimum temperature of YLY6 and HHZ during the major growth stages

Variety	Year	TP-HD	HD-PM	TP-PM	7HD-HD	HD-HD7
YLY6	2012	25.1	19.3	23.3	22.4	24.0
	2013	25.6	21.9	24.3	26.0	25.9
	2014	23.5	20.7	22.4	21.3	23.7
	2015	23.8	19.1	22.0	22.7	21.1
	2016	25.1	19.9	23.3	26.9	21.2
	2017	25.2	19.9	23.1	25.5	23.1
	**Mean**	**24.7**	**20.1**	**23.1**	**24.1**	**23.2**
	**CV (%)**	**3.4**	**5.0**	**3.4**	**9.6**	**7.8**
HHZ	2012	25.3	22.3	24.3	24.9	26.2
	2013	25.4	23.8	24.8	26.7	26.3
	2014	23.9	22.2	23.3	25.1	22.3
	2015	23.9	20.7	22.8	23.8	22.7
	2016	24.7	23.3	24.2	25.9	27.6
	2017	25.2	22.4	24.0	25.7	24.2
	**Mean**	**24.7**	**22.4**	**23.9**	**25.4**	**24.9**
	**CV (%)**	**2.8**	**4.8**	**3.1**	**3.9**	**8.6**

TP-transplanting; HD-heading; PM-maturity; 7HD-the week prior to heading; HD7- the week after heading; CV- coefficient of variation.
